# The “Flexi-Chamber”: A Novel Cost-Effective *In Situ* Respirometry Chamber for Coral Physiological Measurements

**DOI:** 10.1371/journal.pone.0138800

**Published:** 2015-10-08

**Authors:** Emma F. Camp, Sophie-Louise Krause, Lourianne M. F. Santos, Malik S. Naumann, Ruy K. P. Kikuchi, David J. Smith, Christian Wild, David J. Suggett

**Affiliations:** 1 Coral Reef Research Unit, School of Biological Sciences, University of Essex, Colchester, United Kingdom; 2 Coral Reef Ecology Group (CORE), Leibniz Center for Tropical Marine Ecology (ZMT), Fahrenheitstr. 6, 28359 Bremen, Germany; 3 Faculty of Biology and Chemistry (FB 2), University of Bremen, 28359 Bremen, Germany; 4 Coral Reef and Global Changes Research Group (RECOR), Geosciences Institute, Federal University of Bahia, Barão de Jeremoabo St., Ondina University Campus, Salvador, Bahia, Brazil; 5 Functional Plant Biology and Climate Change Cluster (C3), University of Technology Sydney, Broadway, NSW, Australia; James Cook University, AUSTRALIA

## Abstract

Coral reefs are threatened worldwide, with environmental stressors increasingly affecting the ability of reef-building corals to sustain growth from calcification (G), photosynthesis (P) and respiration (R). These processes support the foundation of coral reefs by directly influencing biogeochemical nutrient cycles and complex ecological interactions and therefore represent key knowledge required for effective reef management. However, metabolic rates are not trivial to quantify and typically rely on the use of cumbersome *in situ* respirometry chambers and/or the need to remove material and examine *ex situ*, thereby fundamentally limiting the scale, resolution and possibly the accuracy of the rate data. Here we describe a novel low-cost *in situ* respirometry bag that mitigates many constraints of traditional glass and plexi-glass incubation chambers. We subsequently demonstrate the effectiveness of our novel “Flexi-Chamber” approach via two case studies: 1) the Flexi-Chamber provides values of P, R and G for the reef-building coral *Siderastrea cf*. *stellata* collected from reefs close to Salvador, Brazil, which were statistically similar to values collected from a traditional glass respirometry vessel; and 2) wide-scale application of obtaining P, R and G rates for different species across different habitats to obtain inter- and intra-species differences. Our novel cost-effective design allows us to increase sampling scale of metabolic rate measurements *in situ* without the need for destructive sampling and thus significantly expands on existing research potential, not only for corals as we have demonstrated here, but also other important benthic groups.

## Introduction

Over 60% of coral reefs worldwide are threatened by local stressors such as sediment loading, physical damage, overfishing and pollution, a figure that expands to *ca*. 75% when further accounting for global climatic threats from thermally induced coral bleaching, ocean acidification, and increases in the frequency and magnitude of storm events [1–6]. These various stressors can act in isolation or in concert to negatively impact or completely inhibit the fundamental metabolic processes of reef-building corals, i.e. respiration (R) and photosynthesis (P) [7–10], which together fuel growth via calcification (G) [11–13]. Not only do these physiological processes represent the ‘first response’ to environmental stressors, but also the key processes that govern nutrient cycling and productivity of reef-building corals [13]; as such, researchers have attempted to quantify coral metabolic rates and how they are regulated by key environmental factors for decades [11, 14–18].

At the broadest ecological scale, ecosystem metabolism approaches have proved popular for examining how environmental change influences signatures of metabolism (O_2_ or pH drift) over space (e.g. [19–24]) or time (e.g. [25–27]); however, these approaches are unable to resolve the contribution of individual taxa to the net ecosystem response observed, a problem that must be overcome to understand when/how taxa are differentially influenced by stressors [28–29]. Resolving specific taxonomic contributions requires that individual organisms (e.g. coral colonies/nubbins) must be actively removed and incubated *ex situ* (e.g. [30–32]); however, this practice can be highly destructive and often prohibited by local permitting and also prone to the introduction of experimental artefacts due to poorly replicated *in situ* environmental conditions. An alternate method has been the use of *in situ* benthic chambers, which have been designed with differing levels of complexity (e.g. [33–38]), but all according to the same operational principal. Individual coral specimens are incubated in seawater in sealed transparent vessels installed in the environment for a denoted period of time. Artificial mixing and regular flushing has to be conducted, and either mounted *in situ* sensors record drifts in O_2_ and/or pH, or samples are drawn from the vessels at the start and end of incubation to detect differences [39].

Whilst the *in situ* benthic chamber technique potentially provides a means to better replicate the environmental conditions that ultimately govern key metabolic processes in corals, the design of rigid chambers introduces additional artefacts that inevitably influence the accuracy of metabolic rates returned; most importantly, rigid closed vessels result in loss of water movement that is critical for disruption of the diffuse boundary layer where the water chemistry, nutrient content, and aerobic status around the coral can be very different to that of the surrounding water [39–41]. High-flow speed and/or turbulence inherent to dynamic reef environments reduce the thickness of this boundary layer [42–43] and influences mass flow in corals [44]. In low-flow environments the boundary layer is thicker [45], which in turn reduces passive diffusion and metabolic activity; thus, flow rate is known to positively enhance key physiological processes, notably rates of nutrient uptake [46], P [40], R [47], G [48–49], and growth [49–50]. It therefore stands to reason, that in metabolic studies using sealed chambers, the boundary layer needs to be disrupted to prevent a decrease in passive diffusion and to ensure continued advective exchange. Artificial mixing to chambers has therefore been introduced through the use of stir bars and magnets [51] or via semi-continuous automatic flushing systems [52], but this dramatically increases the technical complexity and sampling effort required.

Secondly, chambers are often of a fixed size, thus a range of chambers may be necessary or experimentation is otherwise limited to a targeted organism size. The size of the chamber (i.e. volume of the incubation medium) relative to the organism to be incubated is critical in order to balance: (i) maximised signal strength as compared to (ii) minimising potential toxicity via super saturation of toxic O_2_ species or anoxic conditions [52]. In shallow environments similar to those where respirometry chambers are most frequently deployed, studies have measured zooxanthellae raising host tissue O_2_ levels to >200% saturation [53–55], and photosynthetically produced O_2_ can be catalysed by UV radiation to produce harmful Reactive Oxygen Species [56].

As part of a study examining the metabolic functioning of shallow water coral communities, we developed a novel *in situ* bag respirometry chamber, the “Flexi-Chamber” to overcome the various limitations of established chambers. Whilst use of a flexible material as an incubation chamber is not entirely novel, e.g. the SHARQ system for community scale assessments [25], we describe here a simple, cost-effective incubation chamber more suitable for targeted and high- throughput benthic metabolic studies. Here we describe and validate the “Flexi-Chamber” method, and report two case studies that demonstrate its application. The first case study compares results of metabolic process measurements (P, R and G) for the coral *Siderastrea*. *cf*. *stellata* at a shallow reef site in Salvador, Brazil, generated by simultaneous deployment of our Flexi-Chambers and an established glass respirometry chamber (see [32]). The second case study demonstrates the wider-scale application of the Flexi-Chambers to measure P, R and G between species and across habitats. We evaluate the effectiveness of this method and discuss the potential that this method has for broader *in situ* experimentation.

## Material and Methods

### Flexi-Chamber description

**Fig 1:**
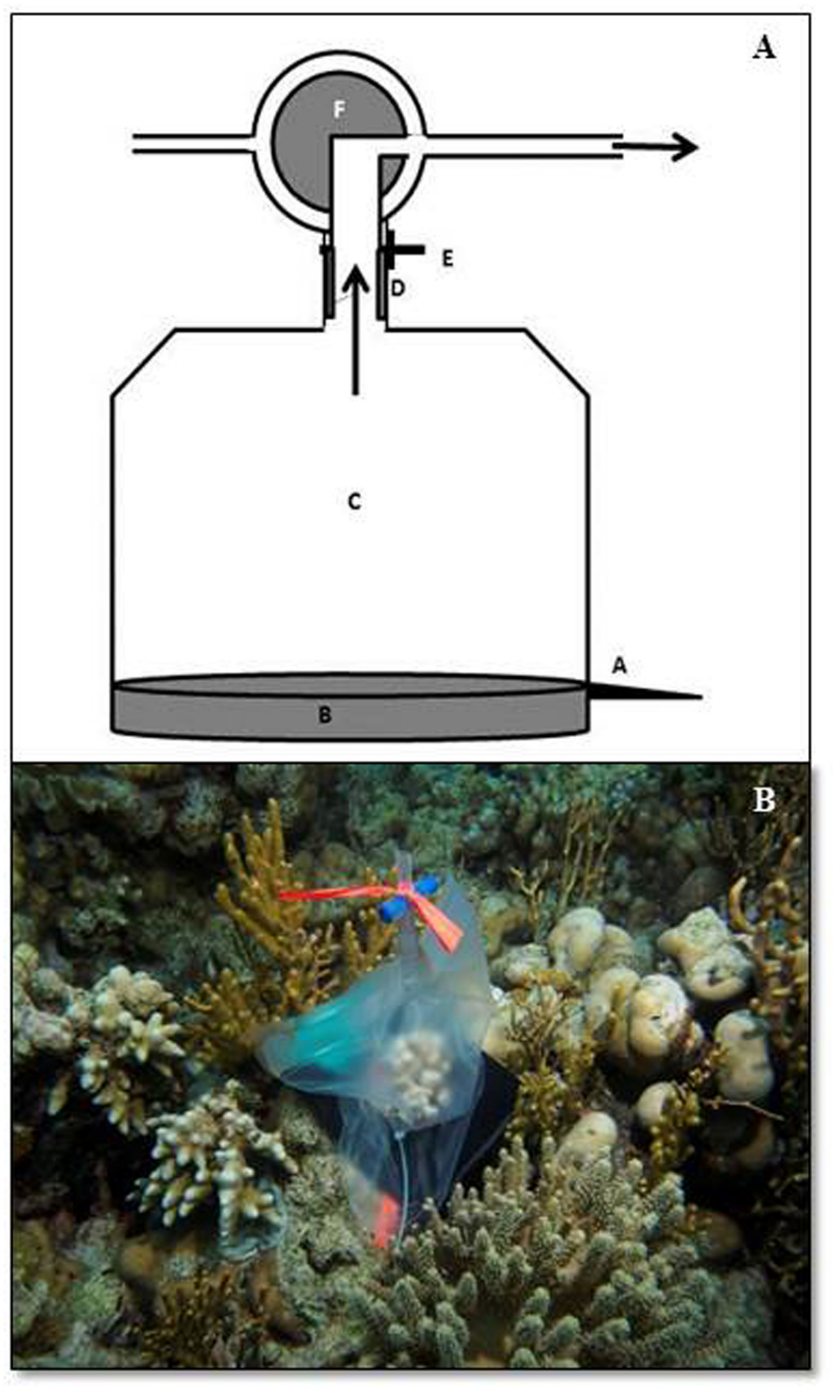
The Flexi-Chamber design. A) Schematic diagram of the Flexi-Chamber: A is the fastening mechanism, B is the neoprene cuff around the base of the coral, C is the urine bag, D is standard silicon tubing used to create a water tight seal with the valve, E is the valve of the urine bag and F is the three-way-valve. B) An example of the Flexi-Chamber set-up.

A transparent, gas-impermeable, 3 L urine bag (Vital Care, Essex, UK) with a built-in heat-seam secured valve, forms the basis of the Flexi-Chamber (Fig 1a). The bottom of the bag is cut to create a fringe that is secured around the base of the test colony. A water-tight seal is created by using a customised neoprene cuff and fastening. The cuff also minimises the possible contribution of the surrounding substrate and/or water column to the metabolic signal of the test specimen (Fig 1b). The internal water volume can be adjusted to accommodate corals of different sizes: larger coral colonies (*ca*. 5 cm diameter) require the entire volume of the chamber, whereas smaller colonies (*ca*. 2–3 cm diameter) require a reduced volume (in this case 60% of the bag volume) to optimise the metabolic signal.

Water is extracted from the chamber via the built-in three-way valve mechanism, which can be opened or closed to sample the internal water volume (Fig 1a). In this way, water can be conveniently extracted via a syringe without cross-contamination of the surrounding seawater. Two 150 ml syringes are attached to the three-way valve system; the first to remove *ca*. 30 ml of excess water from the bag’s valve which is discarded, and the second to extract 100 ml of the seawater sample from inside the Flexi-Chamber. For standardisation of the physiological measurements, water volume inside the chamber was accurately determined as the total of the water volume removed during the sampling process (100 ml) and the remaining water volume within the chamber (typically 950–1000 ml) determined by subsequent syringe removal of water until the bag is emptied. The swivel lock of the syringe to the valves as well as the water tight nature of the bag (see materials and methods: leakage testing) ensured that the water removed from the bag was not contaminated with that from the surrounding seawater.

### Flexi-Chamber incubation procedure

Prior to any experimental use, all Flexi-Chambers were acid washed (2% HCL). For each deployment, three Flexi-Chambers were filled with *in situ* water without any coral colonies to act as a control to correct for any microbial metabolic activity within the surrounding water. Whilst colonies where haphazardly selected within any one habitat, colonies had to be *ca*. 3–5 cm in diameter, and chosen with no visible signs of disease, bleaching, loss of colour (relative to the site mean), or excessive algal overgrowth [57]. Colonies also needed a suitable surrounding substrate to allow the attachment method, i.e. a basal area raised from the substrate for chamber attachment.

Sample water was syringed from each Flexi-Chamber and immediately transferred to pre-labelled 250 ml borosilicate glass bottles [32]. Sample bottles were kept at ambient temperature and under dark conditions until analysis (usually within 1 h of collection [58]). Chambers were left *in situ* for 3 h. After this period, water samples were re-collected from each chamber and stored as previously described. After all samples had been collected, chambers were removed from each colony, the water was flushed with ambient water, and chambers were re-secured; control chambers were treated in exactly the same way. The whole process was then repeated at 3 h intervals for the duration of the study period. Within this study, dark cycle metabolism was examined by artificially darkening (opaque black polyester material) the Flexi-Chambers but the technique could be used equally well during the night time (see [59]).

### Analytical procedures

Salinity of each sample was measured using a handheld refractometer (accuracy within ± 0.5 ppm; model RF20, ExTech, USA). pH was measured using the Metrohm Unitrode combination electrode, calibrated using TRIS buffers (precision *ca*. ± 0.0005 pH units). To determine the O_2_ content (accuracy *ca*. 0.05 *μ*mol/L) of each sample, a 100 ml aliquot of each water sample was transferred into a sealed chamber in the lab, where an O_2_ and temperature probe (O_2_ probe: Foxy-R, Temperature Probe: NeoFox TB; Ocean Optics, England) were attached to a bench-top fluorometer (NeoFox-FT; Ocean Optics, England) via a bifurcated fibre assembly (BIFBORO-10000-2; Ocean Optics, England) and attached to a PC running the O_2_ sensing software (NeoFox Viewer, Ocean Optics). Samples were run in a climate-controlled laboratory until the O_2_ concentrations stabilised. All probes were calibrated according to the Ocean Optics instruction manual.

Total alkalinity (TA) was measured using an open-cell potentiometric titration procedure using the Gran method to determine the second end point of the carbonate system [58]. TA of all samples was determined using a Titrino titrator (model 848; Metrohm, Buckingham, UK). 0.1N standardized HCL was used and TA was measured with an accuracy and precision of *ca*. 2 μmol kg^-1^.

### Measurements of photosynthesis, respiration and calcification

For all samples, G was determined via the TA anomaly method [60] corrected for any changes in TA of the seawater controls, to yield hourly calcification rates (G, mmol CaCO_3_ m^-2^ h^-1^) as:

$$G\;\left(t\right)\;=\left[\frac{\left(\Delta TA\cdot \rho \cdot 0.5\right)\;\cdot V}{I_{t}\;\cdot SA}\right]/1000$$ (1)

Where TA = total alkalinity (*μ*mol kg^-1^), *V* = volume of water (L) surrounding the coral within the respirometry chamber, I_*t*_ (h) is incubation time, SA is the coral surface area (m^2^), *ρ* is the density of seawater and 0.5 accounts for the decrease of TA by two equivalents for each mole of CaCO_3_ precipitated.

Net P and R rates were determined for several time points (*t*) throughout the light and dark cycles respectively as the change in O_2_ in the respirometry chamber corrected for any changes in O_2_ of the seawater controls to yield hourly rates (mmol O_2_ m^-2^ h^-1^) as:

$$P_{N}\;and\;R\;\left(t\right)=\;\left[\frac{\left(\Delta O_{2}\right)\;\cdot V}{I_{t}\;\cdot SA}\right]\;/1000$$ (2)

Integration of all P and R measurements during the light (dark) yielded the daily P_N_ and R (mmol O_2_ m^-2^ d^-1^) as:

$$P_{N}=\sum_{Light-end}^{Light-start}P(t)\Delta t\text{ and }R=\sum_{Dark-end}^{Dark-start}R(t)\Delta t$$ (3)

P_G_ was calculated by the addition of P_N_ and R. All values of *R* are subsequently multiplied by the factor -1 to convert to positive values. Surface area of all colonies was determined by the advanced geometric technique [32].

### Flexi-Chamber method validation

All laboratory testing of the Flexi-Chamber design was conducted using the aquarium facility at the Coral Reef Research Unit, University of Essex, UK (January 2013- December 2014). We used *Acropora sp*. coral specimens supplied by Tropical Marine Centre Ltd. (Chorleywood, UK) as our test organism since this genus is present across bioregions and widely used in physiological process measurements [61]. Five colonies were used and were secured into 1 cm^2^ PVC plugs with a non-toxic epoxy resin (Milliput-Standard) and left to acclimatise for 24 h prior to experimentation. Colonies were not fragmented and therefore did not need to heal before experimentation. Aquarium tanks were supplied with Tropic Marine PRO REEF salt-based seawater supplemented with NaHCO_3_ maintained at 28 ± 0.9°C (using Aquael Neo Heaters IPX8, Poland), 35 PSU, a 4 L min^-1^ flow rate circulating between the tanks and a common biological sump of Fijian live rock (Tropical Marine Centre Ltd., Chorleywood, UK) and were kept under daylight conditions (*ca*. 80.2 *μ*mol photons m^-2^ s^-1^) using 150 W Metal-Halide lamp (Arcadia Products PLC, Redhill, UK). A series of laboratory tests were conducted:

#### Water extraction

To determine the average error of the water extraction method a Flexi-Chamber was filled with exactly 250 ml of seawater and subsequently extracted via the syringe method described above; the volume removed was measured and subtracted from the 250 ml to quantify the amount of seawater unaccounted for. The process was repeated 30 times to gauge the mean and error for this step.

#### Temperature

Possible temperature drifts as a result of incubation were tested. A Flexi-Chamber was filled with synthetic seawater and maintained in an aquarium of a known constant temperature of 28.0 ± 0.9°C (Aquael NeoHeater IPX8, Poland). The internal temperature within the Flexi-Chamber was then determined every hour over an 8 h period by opening the valve and inserting a temperature probe (NeoFox TB; Ocean Optics, England). An *in situ* temperature comparison between the Flexi-Chamber and surrounding seawater was also conducted in Salvador Brazil, with a HOBO Pendant Temperature/Light Loggers (model UA-002-64; Microdaq, USA) set to log every 30 s, placed inside the Flexi-Chamber, and one outside the Flexi-Chamber for three 24 h periods.

#### Water flow

To test the extent of water movement (circulation) within the Flexi-Chamber the relative acceleration in the X, Y and Z planes was determined inside and outside of the Flexi-Chamber during a 1 h period (*n* = 3) using a G Pendant HOBO logger (UA-004-64; Microdaq, USA), set to log every second. The logger had tethered anchored beams in fixed locations that had centre plates. As the beams move, the centre plate is displaced creating a change in capacitance proportional to the applied acceleration [62]. This change in capacitance is converted to an output voltage which is processed with calibration data to produce an equivalent acceleration value where 1G = 9.8m s^-2^. Prior to launch, both loggers were inter-calibrated by comparing their readings relative to one another when moving them through a series of set orientations [62]. Both loggers were initiated at the same time and set to log at 10 s intervals. To establish a reading in all three planes the logger had to be free to move without touching the inside of the chamber. As the logger is positively buoyant, a 6 cm length of cable of 1 mm thickness was used to tie the logger to a weight; resulting in the HOBO being orientated upside down. A small piece of glass was then attached to the underside of the logger to make it neutrally buoyant so that it sat centrally within the Flexi-Chamber. High water velocity could cause the HOBO to make contact with the Flexi-Chamber, constraining its movement and thus the acceleration; however, this was never observed [63] and any restrictions associated with the cabling would influence both HOBOs equally and is therefore negligible. A mesh contour graph was generated to show the location of each HOBO in 3D space over the 1 h incubation time to illustrate the internal and external water movement in the X, Y, Z planes.

#### Lighting

Light penetration through the Flexi-Chamber was measured using a spectroradiometer (M/A-COM, model SR9910-UF; Lamington Scotland), and converted from energy (W m^-2^) to photons (*μ*mol m^-2^ s^-1^) and percent transmission as the amount of light transmitted through the respirometry chamber relative to no chamber present [64]. Artificial lighting (240–800 nm) consisted of LED arrays covering the Photosynthetically Active Radiation (PAR) (400–700 nm) spectra (Heliospectra; Göteborg, Sweden) and UV fluorescent 20 w tubes (Philips, Netherlands) covering UVB and UVA radiation (240–400 nm).

#### Leakage testing

To ensure that a watertight seal could be created around coral colonies, the Flexi-Chamber was secured around a colony of *Acropora sp*. and natural red food colouring was syringed into the Flexi-Chamber via the three-way-valve mechanism. Five replicate colonies were sampled. The Flexi-Chamber was situated inside an aquarium with a pendant temperature/light HOBO placed inside the aquarium and Flexi-Chamber. An initial 30 ml aliquot of water was syringed out of the Flexi-Chamber and also from the surrounding aquarium water. The Flexi-Chamber was left in the aquarium for 3 h, before another water sample was collected from inside the Flexi-Chamber and aquarium. Absorbance of each sample was then measured using a USB 2000^+^ Spectrometer (Mikropack Halogen Light Source (HL-2000), 1 cm Cuvette Holder, serial fibre optic probes (727-733-2447) Ocean optics, England) to determine if any dye had transferred from inside the Flexi-Chamber to the surrounding aquarium water.

#### Gas permeability

Whilst the Flexi-Chamber material is designed to be gas impermeable, we verified negligible permeability for both CO_2_ and O_2_ by filling five Flexi-Chambers with *in situ* seawater (Salvador, Brazil). Mercuric chloride (*ca*. *2* pipette drops, as per CDIAC protocols, [58]) was added to prevent any further biological activity within the water and an initial 100 ml water sample was taken from each Flexi-Chamber. Flexi-Chambers were then secured *in situ* and left for 3 h. After the 3 h period, an additional 100 ml of water was removed from each Flexi-Chamber (end sample). For the initial and end samples taken, O_2_ and CO_2_ were measured. O_2_ was measured using Foxy-R O_2_ probe (Ocean Optics, England) and CO_2_ was measured using a custom-built gas diffusible membrane attached to an external infrared gas analyser [17].

#### Oxygen toxicity

Prior to any incubation procedure, a sensitivity analysis is first required to establish the optimum vessel size-to-organism biomass ratio, relative to the flushing time procedure to prevent anoxic or hypoxic conditions; time taken to reach anoxic or hypoxic conditions will be highly variable as a result of inherent differences in metabolism across taxa and growth environments. A benefit of the Flexi-Chamber is that the internal volume of water can be adjusted to accommodate different volumes of water to help mediate the balance of biomass-to-water required. An example of the sensitivity analysis required and how the Flexi-Chamber can easily be adjusted to accommodate different water volumes was undertaken on *Acropora sp*. 18 colonies of *Acropora sp*. of similar size (mean ± SE) (12.0 ± 0.13 cm^2^) were enclosed in the Flexi-Chambers with a volume of either 250 ml, 500 ml, 750 ml, 1000 ml, 1250 ml or 1500 ml of surrounding seawater to compare when anoxic or hypoxic levels were reached. Chambers were maintained in the aquaria under light-dark cycles (conditions previously described). Initial water samples were collected to measure the O_2_ levels at time zero. An aliquot of 30 ml of seawater was then removed every hour over a 4 h period to examine for changes in [O_2_].

#### Additional stress factors

To ensure that no unforeseen factors were stressing the coral, such as chemicals leaching from the plastic, we further incubated *Acropora sp*. nubbins (*n* = 5) in separate Flexi-Chambers for 9 h with regular 3 h flushing to observe for any visible signs of stress, in the form of mortality, excessive mucus formation or loss of pigmentation. Zooxanthellae counts were taken from tissue stripped from the base of each nubbin [65].

### Flexi-Chamber practical application

#### Case study 1: *In situ* comparison of Flexi-Chamber versus established glass respirometry chamber

Performance of the Flexi-Chamber design was compared *in situ* with that of established glass vessel respirometry routinely used in metabolic activity measurements [32]. Colonies of the commonly occurring *S*. *cf*. *stellata* near Salvador (Brazil) were sampled from the 26^th^ March to the 2^nd^ April 2014. The city of Salvador is located on the South Atlantic east coast of Brazil within the Bahia region. Salvador is situated at the entrance of Todos os Santos Bay (TSB), which is the second largest bay ecosystem of Brazil [66]. The study site was located on a patch of coralline environment at the entrance of TSB in 3–5 m water depth adjacent to the Yacht Clube da Bahia harbour (12°59' S, 38°31' W).

In total 40 colonies of *S*. *cf*. *stellata* were examined for P, R and G throughout a one-week period. All incubations occurred at a water depth of *ca*. 2–3 m. For 30 of the colonies, measurements using the Flexi-Chamber and glass chamber were made on separate colonies (15 colonies per chamber type). For the remaining ten colonies, metabolic measurements for both chambers were made on the same colony but for different days, with a minimum rest period of 24 h for any one colony between measurements, and random allocation to the initial chamber.

Colonies tested in the glass chamber were removed from the seafloor at least 48 h prior to measurements [32]. During collection all coral colonies were handled without any air exposure or direct tissue contact and gloves were worn throughout the handling process. Any extensive epibionts or endolithic boring organisms were excluded and any remaining overgrowth was cleaned using a soft toothbrush. Similarly, any air bubbles or large particulates in the water were removed from the chambers, to minimise O_2_ fluxes from non-coral sources [57]. Colonies were chiselled from their substrate and transferred using individual zip-lock bags to avoid mechanical damage during transport [32]. The chambers were fixed to a custom made metal frame, to facilitate transport and rapid deployment *in situ*. Chamber incubations were conducted under no-flow conditions so hourly manual stirring (magnetic stirring bars) was necessary to break-up the boundary layer [34].

In this case study, a 3 h light incubation was followed by a 3 h dark incubation. All incubations were run around the daylight maximum (*ca*. 11:00–14:00) for the light incubations for P_N_ and G_L_. Corresponding dark rates, i.e. R and G_D_, were obtained during daylight hours by covering the Flexi-Chambers with an opaque black polyester material bag. During the dark sampling periods, the Flexi-Chambers were left for 1.5 h before the 3 h sampling session to ensure steady state respiration rates that were consistent with prolonged maintenance in darkness.

#### Case study 2: Broad scale deployment of Flexi-Chambers

To demonstrate the broad scale application of the Flexi-Chambers across sites and between species, three coral species: *Mussismilia harttii*, *S*. *cf*. *stellata* and *Porites astreoides*, were examined for metabolic activity (P, R and G) at two coastal sites on the island of Morro de São Paulo, Bahia, Brazil. Both sites were lagoon environments situated in *ca*.1-2 m water depth. Sampling took place between the 20^th^ and the 25^th^ of February 2014. In total, five individuals of each species were examined within each site over five consecutive days. A HOBO Pendant Temperature/Light (Lux) Logger was installed within each habitat to log every 30 s. Salinity was measured at the end of each incubation. The northern site was located in close proximity to shore (13°22' S, 38°54' W) and experienced more variable and extreme conditions (e.g. greater fluctuation in salinity, temperature and light). The daily temperature range was 4.0 °C and high-turbidity resulting from strong wave action was likely responsible for the lower average irradiance (10003.5 ± 450 lux). Regular fresh-water run-off after rainfall affected the northern site and accounted for a daily range in salinity of 4.5 ppm. The second site was located in a more southerly bay along the coast and was situated further offshore (13°23' S, 38°54' W). The southern site had similar mean temperature to the northern site (28.6 ± 0.04 °C) but a smaller temperature range (2.9 °C). The increased distance of the southern site from land meant that freshwater impacts were reduced, with mean salinity significantly elevated to the northern site (35.3 ± 0.03 ppm, *t*_121_ = -3.30, *P* = 0.001) and a smaller range (1.5 ppm). The southern site also experienced less turbidity likely due to the wave action dissipated by a shallower fringing reef, which resulted in significantly elevated average irradiance relative to the northern site (15732.1± 411.5 lux, *t*_674_ = -3.18, *P* = 0.002); however, it still experienced large tidal cycles common to the Bahia region (1.1 m at neap tide up to 2.6 m at spring tide during the sampling period).

At both sites on the coast of Morro de São Paulo, one colony of each species (*M*. *harttii*, *S*. *cf*. *stellata* and *P*. *astreoides*) was incubated in a separate Flexi-Chamber along with three seawater controls, and measurements of P, R and G were determined. Colonies were incubated, and metabolic measurements calculated as detailed for the Flexi-Chamber, repeated over five consecutive days. Incubations were conducted over two 3 h cycles: one light and one dark cycle *as per* case study one.

### Statistical analysis

#### Laboratory validation

Water velocity within the Flexi-Chamber was measured in three planes: X, Y and Z and compared inside and outside of the Flexi-Chamber using a t-test in SPSS 17. Paired t-tests were used to compare the start and end O_2_, CO_2_, mean Hobo light data, and zooxanthellae densities to assess whether there were any differences between the start and end of incubations. The test assumptions for homogeneity of variance (Levine’s test) and normal distribution (Sharpiro-Wilk test) of the data were met. To test the integrity of the watertight seals, start and end absorbance for inside the Flexi-Chamber and in the aquarium were compared using a Pearson’s correlation conducted in R software [67]. Similarly, temperature inside the respirometry chamber was compared to the surrounding water *in situ* using a Pearson’s correlation.

#### Case study 1

For the ten *S*. *cf*. *stellata* colonies whose P, R and G were compared using both chamber designs, a paired t-test was used to compare each metabolic variable. Differences in mean P, R and G for the remaining 30 *S*. *cf*. *stellata* were compared between the two respirometry chamber designs using an independent t-test.

#### Case study 2

Daily P_N_, R, and net G were each compared across species and habitats using Linear Mixed Effects (LME) models, with day as a random effect. Before model application, data was explored (as per [68]). Cleveland dot-plots were used to determine if there were any outliers, and boxplots and scatterplots were used to check for co-linearity. Assumptions of linearity, independence, homoscedasticity and normality were met. The model was fitted using the lme function in the nlme package in R software [69]. Model simplification was undertaken using ANOVA to compare models with progressively simplified fixed effects; thus ensuring correct *p* values [69]. The acceptability of the model was tested by plotting the residuals against: a) the fitted values to check for homogeneity and b) each explanatory variable in the model and dropped during model selection to check for violations of independence [70]. Parameter estimation in lme models was done based on Restricted Maximum Likelihood (REML). The final model applied was:

CJ = α + β1* RespirometrychamberJ + εJ (4)

Where C is metabolic parameter being measured, J is the day, α is the intercept, β is the slope of the respirometrychamber and ε_J_ is the residual error.

## Results

### Flexi-Chamber method validation

#### Water extraction

A 0.5% error was induced onto the metabolic rate (P, G, R) measurements per ml of water not re-extracted from the Flexi-Chamber. From the 30 replicates, the range of water volumes unaccounted for in the syringe removal ranged from 0.5–5.0 ml of water, with an average of 2.0 ± 0.4 ml. Consequently, the average error of the system is 1% but never >2.5%), based on the water extraction procedure.

#### Temperature

Temperature for the first 3 h of the 8 h laboratory test remained within 0.1°C of the ambient temperature; however, after 4 h the temperature within the Flexi-Chamber started to rise considerably, with a 1.4°C increase relative to the surrounding water after 8 h. The temperature of the Flexi-Chamber *in situ* exhibited high co-variability with the surrounding water temperature (*r* = 0.99, *n* = 947, *P* = 0.001). There was no measurable temperature difference between the Flexi-Chamber and *ex situ* water during the day; however, during the hottest period of the day (12.00–17.00 h) the temperature in the Flexi-Chamber increased up to 0.3°C greater than the surrounding water; thus highlighting the need to regularly flush the Flexi-Chambers.

#### Water Flow

**Fig 2:**
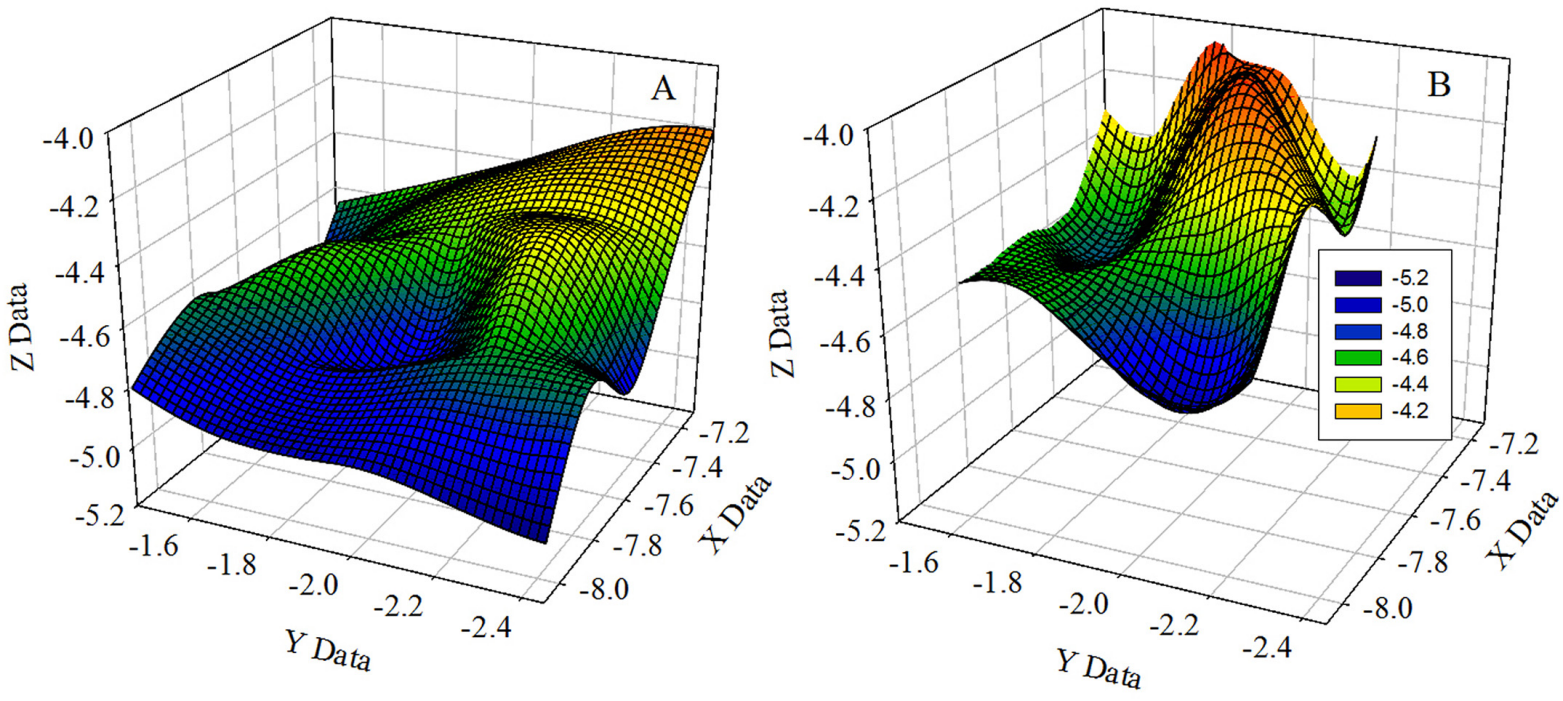
Mesh-contour graphs comparing the water acceleration (m^-2^ s^-1^) along the X, Y and Z planes inside and outside the Flexi-Chambers. The graphs show the location of each HOBO in 3D space over the 1 h incubation time. At each logging interval (1 s) an X, Y, Z coordinate was generated and is plotted onto the graph: (A) shows the external water acceleration, and (B) shows the water acceleration within the Flexi-Chamber measured with a Hobo Pendant G data logger.

Overall, water acceleration along the three axes (X, Y and Z) within the Flexi-Chamber was between 78.0–80.1 ± 2% of the surrounding water acceleration (Fig 2). Despite the overall reduction in water acceleration experienced inside the Flexi-Chamber, there were no detectable differences between each individual plane of movement (Fig 2). The undulations visible on Fig 2b represent periods of restricted water movement within the Flexi-Chamber relative to the ambient water which is represented by a smoother contour in Fig 2a. Greatest loss of movement was in the X dimension due to the way in which the HOBO logger was tethered and the Flexi-Chamber fixed to the substratum.

#### Light

**Fig 3:**
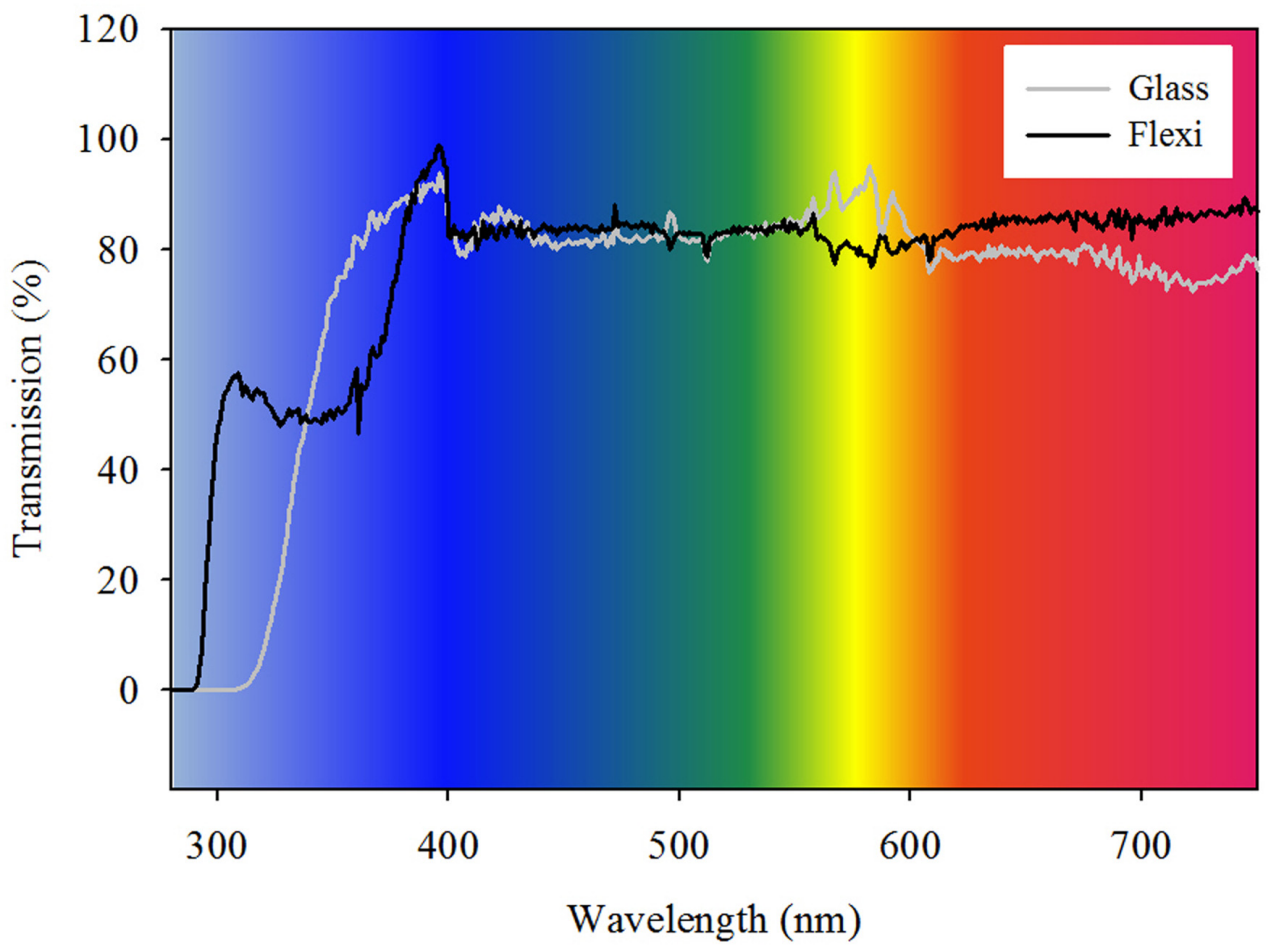
The percent light transmission through the Flexi-Chamber and a traditional glass respirometry chamber. Transmission was tested using a spectroradiometer for PAR and UVB and UVA light. Light was converted from energy (W m^-2^) to photons (μmol m^-2^ s^-1^) (see main text), and percent transmission determined as the amount of light transmitted through the respirometry chamber relative to no chamber present.

Light transmission through the Flexi-Chamber was independent of wavelength within PAR and averaged 84% (Fig 3). However, there was greater loss of light within the UV range (64% and 30% for UVA and UVB respectively).

#### Leakage testing

A strong correlation between start and end absorbance both inside and outside of the Flexi-Chamber suggested none-to-minimal transfer of dye between the Flexi-Chamber and surrounding aquarium water (*r* ranging from 0.24–0.20, *n* = 2048 per replicate, *P* = 0.01); thus confirming that a water tight seal was created.

#### Gas permeability

No detectable difference was measured in the mean (± SE) O_2_ (168 ± 0.2 *μ*mol) and CO_2_ (321 ± 11 ppm) levels inside the chamber for any of the replicates over the 3 h incubation period.

#### Oxygen toxicity

Hypoxia occurred for all colonies kept within 250 ml of water within 1 h of sampling (Table 1a). Subsequent hourly sampling showed that all samples surrounded by 500 ml of water showed hypoxia at 2 h (and 750 ml at 3 h). For the volumes that were not super-saturated by 3 h (1000 ml, 1250 ml and 1500 ml), a detectable change in metabolism was identified. As with hypoxia trends, anoxic conditions were observed under dark conditions for all colonies incubated in 250 ml and 500 ml of water within 1 h (and two colonies incubated in 750 ml anoxic by the 3 h) (Table 1b). From these results, it was established that for *Acropora sp*. at the biomass used, an optimum volume of 1000–1250 ml of water was necessary to ensure a detectable change in metabolic response without hypoxia during daylight hours or anoxic conditions during darkness.

**Table 1:** A) Oxygen hypoxic conditions (O_2Sat_) and detectable metabolic change (Δ_Met_) for internal water volume of the Flexi-Chamber during light conditions for *Acropora sp*. B) Oxygen anoxic conditions (O_2Sat_) and detectable metabolic change (Δ_Met_) for the internal water volume of the Flexi-Chamber during dark conditions for *Acropora sp*.

**1.a**									
	**Water sample collection (Time in hours)**								
	**1**		**2**		**3**		**4**		
**Volume of water within the Flexi-Chamber**	**O**_**2**Sat_	**Δ**_Met_	**O**_**2**Sat_	**Δ**_Met_	**O**_**2**Sat_	**Δ**_Met_	**O**_**2**Sat_	**Δ**_Met_	**Decision**
250 ml	+	+	+	+	+	+	+	+	X
500 ml	±	+	+	+	+	+	+	+	X
750 ml	±	+	±	+	+	+	+	+	X
1000 ml	–	±	–	+	**–**	**+**	±	+	✓
1250 ml	–	+	–	±	**–**	**+**	–	+	✓
1500 ml	–	–	–	±	–	±	–	+	X
**1.b**									
250 ml	+	+	+	+	+	+	+	+	X
500 ml	+	±	+	+	+	+	+	+	X
750 ml	–	+	–	+	±	+	+	+	X
1000 ml	–	±	–	+	**–**	**+**	±	+	✓
1250 ml	–	–	–	±	**–**	**+**	–	+	✓
1500 ml	–	–	–	–	–	–	–	±	X

Results are based on three colonies of *Acropora sp*. (surface area *ca*. 12 cm^2^) at each water volume. O_2Sat_ is whether the water is hypoxic (1.a) or anoxic (1.b) in oxygen and Δ_Met_ is whether there is a detectable metabolic change (total alkalinity and oxygen). The—indicates a unanimous negative response among the three coral replicates, i.e. no hypoxic/anoxic conditions or no detectable metabolic change, ± indicates a mixed response within the three colonies tested. Finally, + indicates a unanimous positive response, so all colonies hypoxic/anoxic or all colonies showing a measurable metabolic change.

#### Additional stress factors

No visual signs of stress (excessive mucus release, loss of pigmentation) were detected for any of the colonies and zooxanthellae concentration (3.5 ± 0.7 x 10^6^ cells cm^-2^) remained similar between the beginning and end of the incubation.

### Flexi-Chamber practical application

#### Case study 1

The Flexi-Chamber and glass chamber had comparable internal temperature, visible light transmission and UVA light permeability, but not UVB light penetration or internal water movement. The Flexi-Chamber acted as a neutral density filter for the major wavelengths of blue, green and red and allowed 50% more transmission of UVB, compared to the glass chamber, which exhibited relatively reduced transmission in the red (Fig 3: Blue: 82.2%, Green: 84.3%, Red 77.3%). Furthermore, the Flexi-Chamber provided consistent water movement within the chamber (78.0–80.1 ± 2% of the external water movement), whereas the glass chamber had zero water flow until the one minute continuous hourly stirring was applied (89.2–131.0 ± 4% of the external water movement).

**Fig 4:**
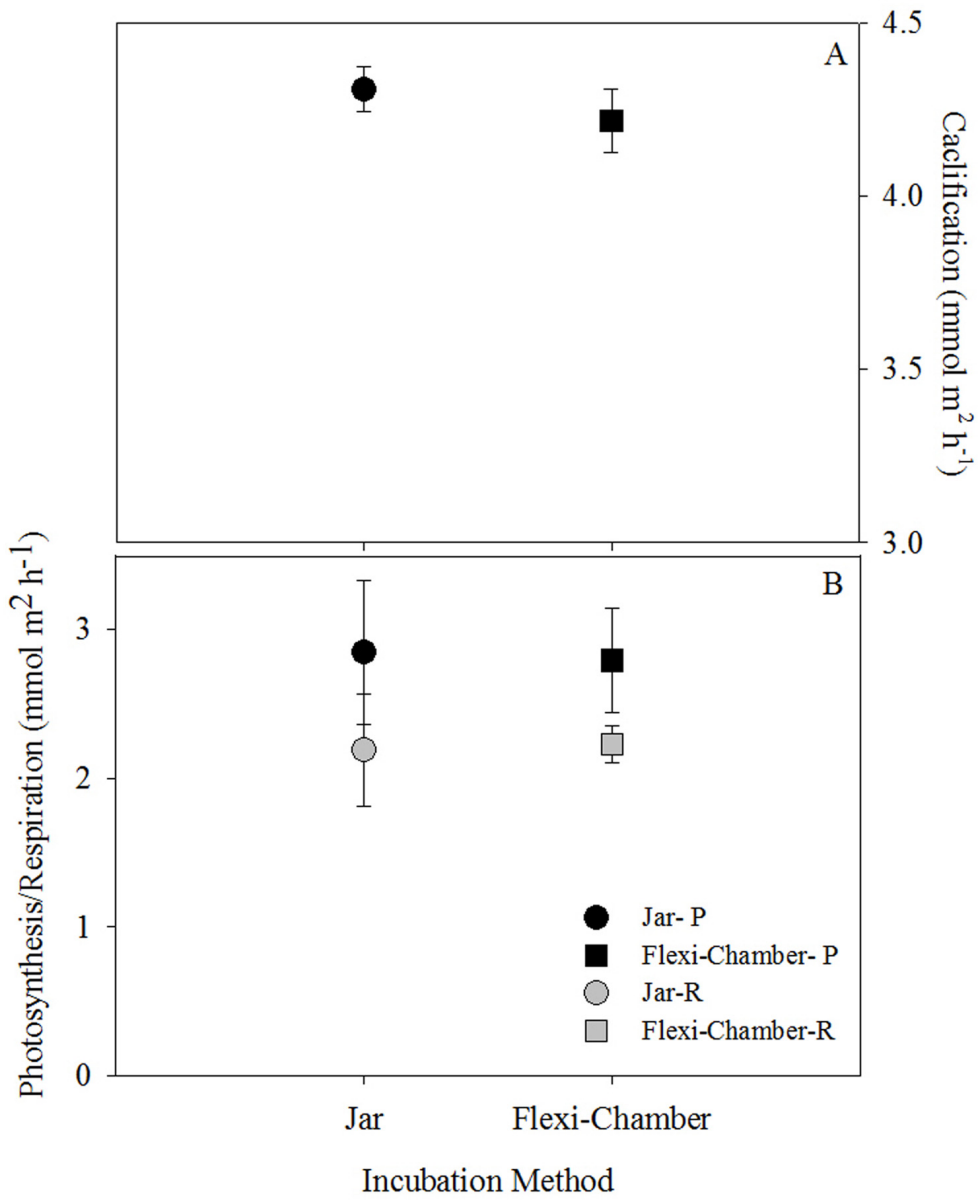
Physiological measurements for *S*. *cf*. *stellata* in Salvador Brazil using a conventional glass chamber and the Flexi-Chamber. (A) Mean calcification (G) rates and (B) mean photosynthesis and respiration rates for 30 colonies of *S*. *cf*. *stellata* are plotted with standard error.

Values for P, R and G showed no significant statistical difference for the glass and Flexi-Chamber across the ten replicate colonies of *S*. *cf*. *stellata* (Fig 4). Similarly, the metabolic rates obtained for the 15 colonies measured in the Flexi-Chamber versus those from the 15 colonies measured in the glass chamber were not statistically different.

#### Case study 2

**Fig 5:**
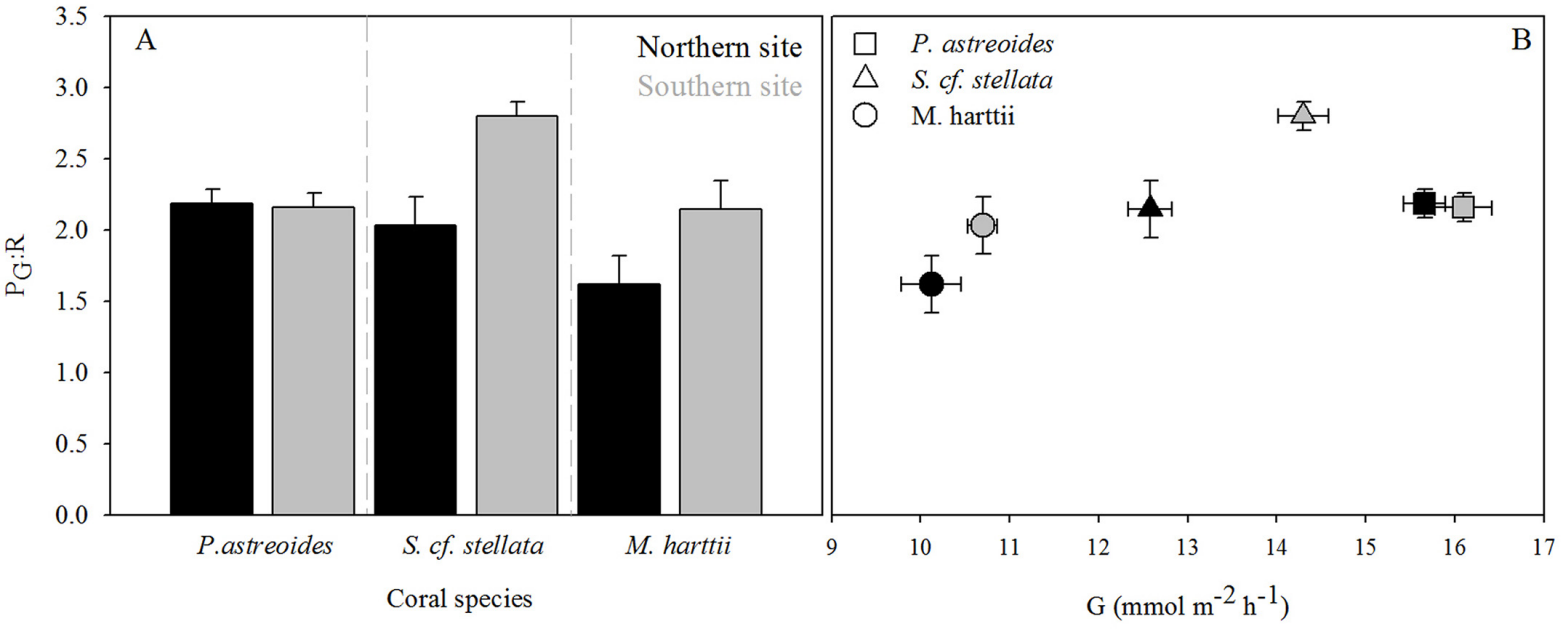
Physiological measurements for corals in Morro de São Paulo, Bahia, Brazil. The ratio of P_G_ to R (panel A), and P_G_ to R ratio plotted against daily G (panel B) for three coral species: *P*. *astreoides*, *S*. *cf*. *stellata* and *M*. *harttii* for two lagoon study sites. Means are shown with standard error (*n* = 5).

The comparison of *M*. *harttii*, *S*. *cf*. *stellata*. and *P*. *astreoides* between the northern and southern lagoon sites on Morro de São Paulo revealed that P_G_ (mean ± SE) was higher in the southern site for *S*. *cf*. *stellata* and *M*. *harttii* (Fig 5a, Table 2b: south site: *S*. *cf*. *stellata*: 8.4 ± 0.2, *M*. *hartti*: 5.8 ± 0.1, north site: *S*. *cf*. *stellata* 6.5 ± 0.2, *M*. *hartti*: 4.7 ± 0.1 mmol m^-2^ h^-1^; *P* = 0.001 and 0.01 respectively), whilst values of P_G_ for *P*. *astreoides* did not change across sites (north site: 11.4 ± 0.1, south site: 12.3 ± 0.2 mmol m^-2^ h^-1^). In contrast to P_G_, R did not change for any taxa between sites, and P_G_:R generally followed the trend of G (Table 2a). Rates of G were highest for *P*. *astreoides* across both sites (north: 15.7 ± 0.2, south: 16.1 ± 0.3 mmol m^-2^ h^-1^). Importantly, P_G_:R correlated with changes in G, except for *P*. *astreoides* where G remained unchanged between sites (Fig 5b).

**Table 2:** A) The metabolic (Net photosynthesis (P_N_), Gross photosynthesis (P_G_), Respiration (R) and Calcification (G)) activity of three coral species at two sites on the coast of Morro de São Paulo, Bahia, Brazil. Incubations were conducted in March 2015 on five colonies of each species over five replicate days. Data are averages (*n* = 5) and standard error. Rates are mmol m^-2^ h^-1^. B) Model parameters to estimate photosynthesis, respiration and calcification as a function of habitat and coral species with day modelled as a random effect.

**2.a**					
**Site**	**Coral**	**P**_**N**_	**P**_**G**_	**R**	**G**
**Northern**	*P*. *astreoides*	6.4 ± 0.3	11.4 ± 0.1	5.0 ± 0.2	15.7 ± 0.2
	*S*. *cf*. *stellata*	3.3 ± 0.3	6.5 ± 0.2	3.2 ± 0.1	10.7 ± 0.2
	*M*. *harttii*	1.8 ± 0.4	4.7 ± 0.1	2.9 ± 0.2	10.1 ± 0.3
**Southern**	*P*. *astreoides*	6.6 ± 0.6	12.3 ± 0.2	5.7 ± 0.1	16.1 ± 0.3
	*S*. *cf*. *stellata*	5.4 ± 0.2	8.4 ± 0.2	3.0 ± 0.1	14.3 ± 0.3
	*M*. *harttii*	3.1 ± 0.6	5.8 ± 0.1	2.7 ± 0.3	12.7 ± 0.2
**2.b**					
**Metabolic Parameter**	**Model Term**	**Estimate**	**SE**	***t*-value**	***P*-value**
**Net Photosynthesis**					
	**Intercept-Species (*M*. *harttii*)**				
	*S*.*cf*. *stellata*	0.66	0.21	3.09	0.01
	*P*. *astreoides*	6.08	0.21	28.45	0.001
	**Intercept-Species (*P*. *astreoides*)**				
	*S*. *cf*. *stellata*	-7.18	0.36	-19.84	0.001
	**Intercept-Habitat-Northern site**				
	*S*. *cf*. *stellata*	2.32	0.10	24.28	0.0001
	*P*. *astreoides*	0.14	0.10	0.86	0.40
	*M*. *hartii*	1.50	0.10	15.70	0.001
**Gross Photosynthesis**					
	**Intercept-Species (*M*. *harttii*)**				
	*S*.*cf*. *stellata*	1.14	0.36	3.15	0.005
	*P*. *astreoides*	8.32	0.36	22.99	0.001
	**Intercept-Species (*P*. *astreoides*)**				
	*S*. *cf*. *stellata*	-5.42	0.21	-25.36	0.01
	**Intercept-Habitat-Northern site**				
	*S*. *cf*. *stellata*	2.12	0.16	13.10	0.001
	*P*. *astreoides*	0.14	0.16	1.47	0.16
	*M*. *hartii*	1.36	0.16	8.40	0.01
**Respiration**					
	**Intercept-Species (*M*. *harttii)***				
	*S*.*cf*. *stellata*	0.32	0.16	1.97	0.06
	*P*. *astreoides*	2.56	0.16	15.74	0.01
	**Intercept-Species (*P*. *astreoides)***				
	*S*. *cf*. *stellata*	-2.24	0.16	-13.77	0.001
	**Intercept-Habitat-Northern site**				
	*S*. *cf*. *stellata*	0.01	0.08	1.25	0.25
	*P*. *astreoides*	0.04	0.07	0.55	0.56
	*M*. *hartii*	-0.02	0.07	-0.27	0.78
**Calcification**					
	**Intercept-Species (*M*. *harttii)***				
	*S*.*cf*. *stellata*	-0.24	0.84	-0.29	0.78
	*P*. *astreoides*	7.40	0.84	8.85	0.0001
	**Intercept-Species (*P*. *astreoides)***				
	*S*. *cf*. *stellata*	-7.64	0.84	-9.14	0.001
	**Intercept-Habitat-Northern site**				
	*S*. *cf*. *stellata*	3.28	0.37	8.78	0.001
	*P*. *astreoides*	0.60	0.37	1.61	0.12
	*M*. *hartii*	2.46	0.37	6.58	0.001

## Discussion

Key to furthering our knowledge of the future of reef building species is to gain a much better understanding of the combined impacts of the different environmental conditions influencing corals within the environment [4, 6]. To do this it is imperative that we are able to accurately and precisely assess biological processes central to the ecological success of species that are sensitive to changing environmental conditions, including multiple disturbance events. This can be achieved by establishing: 1) what is the baseline metabolism (P, R) and G of dominant reef building species? 2) How these physiological processes are altered in response to a change in environmental conditions either combined or in isolation? And consequently, 3) whether we can predict the biological “costs” of future environmental change to the main reef forming species and ecosystem architects? One way to address these questions is to deploy cost effective *in situ* respirometry chambers across species and environments thereby encapsulating the high degree of variability that exists within the natural environment disturbed or not [33–38]. This is particularly challenging in areas where permitting is restricted and thus, the collection or use of colonies is prohibited. Here we have demonstrated that robust metabolic data can be acquired that is at least equal in performance to traditional glass or plexi-glass chambers, but instead using low-cost and easily deployable Flexi-Chambers.

### Flexi-Chamber method advantages

A major advantage of the Flexi-Chamber is the opportunity it provides to establish relatively “high throughput” and therefore, large scale replication with high temporal and spatial resolution without the need for large and costly infrastructure in deployment and sampling. Within this research program we examined the metabolic characteristics of shallow water coral communities by deploying 30 Flexi-Chambers during one 3 h sampling session with minimal cost and effort. An additional advantage of the Flexi-Chamber system is the three-way-valve mechanism enabling additions and removals to be made to and from the sample thereby allowing the chamber water chemistry to be manipulated (e.g. carbonate chemistry or nutrient conditions) without extraction of the coral sample. Although this study has focused on reef building corals, the Flexi-Chamber can potentially be applied to any benthic autotroph.

Another important advancement of the Flexi-Chamber is the ability to adjust the internal water volume to optimise the metabolic signal for the coral biomass selected and incubation, and hence flushing time used. The *Acropora sp*. sensitivity analysis demonstrated the importance to initially establish the correct water volume to biomass ratio and incubation time for any given incubation configuration. Before full and final experimental application of the Flexi-Chamber, users should identify the optimal time, coral biomass, and water volume, through such sensitivity analysis to ensure anoxic or hypoxic conditions do not occur for their organism or growth environment of interest. Differences in *Symbiodinium* spp., coral heterotrophy-to-autotrophy balance and local abiotic and/or experimental conditions all have the potential to influence metabolic rates and thus the time taken for anoxic or hypoxic conditions to occur.

### Method Comparison

An important outcome of the study is the similarity between the data returned from the Flexi-Chamber when compared to traditional and conventional glass chambers [32]. Consequently datasets obtained by the two techniques can be compared historically or in the future; thus implementing this new method development does not preclude consideration of longer terms trends in the metabolic function of reef building corals or other benthic organisms. The comparison of the glass and Flexi-Chamber identified differences in their physical properties; however, despite these differences they still provided similar metabolic rates suggesting that any inherent limitations carry an equal amount of inaccuracy. Whilst we do not have a measurement to benchmark this inaccuracy to either approach, our results suggest that the error between approaches is lower than the differences expected between organisms. Further research is needed to benchmark these methods against a ‘gold standard’ that can fully replicate the *in situ* environment.

### *In situ* application

Our field deployment of the Flexi-Chamber provided novel metabolic data from Brazil’s marginal coral reefs, including the endemic species *M*. *harttii*. In the northern lagoon site, which experienced lower light intensities and salinity, *M*. *harttii* and *S*. *cf*. *stellata* showed decreased G corresponding to the light driven reduction in P [71]. *P*. *astreoides* maintained G between the two sites despite the difference in environmental conditions as P and R also remained constant. Without further experimentation it is hard to currently explain the very different physiological response of *P*. *astreoides*; however, our results confirm that corals are able to expand their niche and inhabit a wider range of environments through adjustment of their metabolism to a wide range of conditions [72]. The results of case study two demonstrate that the Flexi-Chamber can be used to measure inter- and intra-species differences. No prior studies have investigated the physiology of the coral species in the Bahia region, but our G data is similar to values found in other locations [13, 34].

### Flexi-Chamber advancement

Despite the benefits of the Flexi-Chamber there are still some limitations: (i) Only 84% of incident PAR and 54% of UV is currently transmitted and it may be possible to source other materials to enhance light transmission; (ii) Some small error is induced from the water extraction methods; (iii) The current 3 L maximum volume of the Flexi-Chamber and the existing attachment method limits the type of colony that can be examined. Although this current volume limitation is similar to traditional glass chambers, there are options to significantly increase the Flexi-Chamber through the production of bespoke bags. This would be an important advancement in metabolic analysis since current respirometry methods are limited to smaller colonies which do not represent the bulk of coral biomass in most classical reef systems [73]. For example, colonies that have reached a size where they become reproductively active may well have different metabolic characteristics compared to newly formed smaller colonies. It remains to be seen whether the improvements of the Flexi-Chamber identified here are scalable. The flexibility of the system means that further development could enhance the system, for example tubing to the surface for sample collection in shallow environments to remove the need for snorkelling/diving. The Flexi-Chamber could also be advanced by the addition of a plasticiser depending on experimental needs. These developments should not be considered as a limitation to the existing approach but should rather highlight the advantages to such a flexible system.

## Conclusion

This study demonstrates that near off-the-shelf materials can be used to produce cheap and reliable chambers that can be deployed across a range of habitats in a very cost effective manner. The Flexi-Chamber offers an immediate sampling solution that over-comes some of the engineering and logistical challenges of conventional respirometry chambers by allowing non-destructive, *in situ* metabolic analysis. The ability to adjust the Flexi-Chambers internal volume and the natural mixing ability of the chamber provides two important benefits of this design. The comparative metabolic results of *S*. *cf*. *stellata* in Salvador Brazil, between a conventional glass vessel demonstrates that the chamber design described here offers an alternative respirometry chamber for coral metabolic studies, with great potential to be developed further for other test organisms and applications.
